# Addressing constraints to informal providers’ involvement in tuberculosis control: a qualitative study of patent medicine dealers and tuberculosis programme managers

**DOI:** 10.1186/s41256-021-00227-x

**Published:** 2021-11-08

**Authors:** Cosmas Kenan Onah, Benedict Ndubueze Azuogu, Edmund Ndudi Ossai, Adaoha Pearl Agu, Victoria Chioma Azuogu, Isaac Alobu, Emeka Onwe Ogah

**Affiliations:** 1 Department of Community Medicine, Alex Ekwueme Federal University Teaching Hospital, Abakaliki (AEFUTHA), P.M.B: 102, Abakaliki, Ebonyi State Nigeria; 2 Department of Community Medicine, College of Health Sciences, Ebonyi State University, Abakaliki, Nigeria; 3 African Institute for Health Policy and Health Systems, Ebonyi State University, Abakaliki, Nigeria; 4 Department of Nursing Services, AE-FUTHA, Abakaliki, Ebonyi State Nigeria; 5 National Tuberculosis, Leprosy and Buruli Ulcer Control Programme, Ebonyi State Ministry of Health, Abakaliki, Nigeria; 6 Department of Paediatrics, AE-FUTHA, Abakaliki, Ebonyi State Nigeria

**Keywords:** Tuberculosis control, Patent medicine dealers, Chronic cough, Presumptive tuberculosis referral, DOTS, Nigeria

## Abstract

**Background:**

A major constraint to tuberculosis control is low case finding with under-reporting to national authorities. Evidence shows that Patent Medicine Dealers are first port of call for most people with symptoms of tuberculosis, yet there is poor referral of such clients to tuberculosis treatment facilities for further evaluation. This study investigated constraints to involvement of Patent Medicine Dealers in tuberculosis control.

**Methods:**

This was a cross-sectional qualitative study among Patent Medicine Dealers and Tuberculosis Control Programme Managers in Ebonyi State Nigeria. Sixty-four Patent Medicine Dealers and five Tuberculosis Control Programme Managers were interviewed using Focus Group Discussion and In-Depth Interview respectively. Data was collected with electronic audio-recording device and analyzed using thematic approach.

**Results:**

There are some knowledge gaps about tuberculosis signs, symptoms, free-treatment policy and mode of operation of care service among Patent Medicine Dealers. Patent Medicine Dealers and Tuberculosis Control Programme Managers are willing to collaborate in tuberculosis control effort but constant demand for incentives by Patent Medicine Dealers and inability of National Tuberculosis Control Programme to keep up with such demands are obvious constraints.

**Conclusions:**

Knowledge gaps in tuberculosis, its control, constant demand for incentives by Patent Medicine Dealers and inability of National Tuberculosis Control Programme to satisfy such demands are constraints to involvement of Patent Medicine Dealers in tuberculosis control. More robust engagement of Patent Medicine Dealers in tuberculosis control with clear job description through tuberculosis education and provision of incentives to support them are recommended policy approaches to improve linkage of clients to tuberculosis treatment facilities.

## Background

Tuberculosis (TB) is one of the top 10 causes of death worldwide and the leading cause of death from a single infectious agent [1]. Everyday, close to 4000 people die from TB and about 30,000 fall ill with the disease [2]. In 2019, the World Health Organization (WHO) estimated that 1.4 million people globally died of TB out of 10.0 million who fell ill with the disease [1]. Prompt diagnosis of TB is a priority for its control and the key step to diagnosis is referral of Presumptive TB Clients (PTBCs) to a Directly Observed Treatment Short-course (DOTS) facility where definitive diagnosis and treatment take place.

A major constraint to TB diagnosis and treatment is inadequate screening and referral of clients with chronic cough. Evidence suggests involving all care providers used by PTBCs is necessary to increase case detection, facilitate diagnosis and reduce inappropriate treatment, drug resistance and financial burden on patients [3, 4]. Documented evidence shows that in low- and middle-income countries, Patent Medicine Dealers (PMDs) who largely operate pharmacies and drug shops constitute the first port of call for most persons with symptoms of TB [5–13], and provide medicines and services for other health needs. Accordingly, PMDs are indispensable to TB control and their potential role in this regard has been reported [8, 9, 11–14].

Nigeria is a high TB-burden country [1] with many and equitably distributed PMDs across her states [15, 16]. Recent WHO TB reports show that the country has recorded one of the lowest TB case detection globally [1, 17–22]. The PMDs like other informal health service providers, are often not linked to public sector-based National Tuberculosis and Leprosy Control Programme (NTBLCP) that implement DOTS [23]. In line with the WHO End TB Strategy which emphasizes engagement of private care providers in TB programmes [24], and WHO’s recommended approaches for early detection of TB [25], the need to engage PMDs to scale up TB case detection cannot be overemphasized.

Patent Medicine Dealers are a great opportunity for TB control because of their huge clientele of PTBCs. They are also a threat to TB control efforts due to their limited training, poor diagnostic skill and limited patient treatment capability [6, 26], and poor client referral to higher level of health care [6, 8, 9, 27]. Hence, this study investigated the constraints to PMDs’ involvement in TB control to guide policy-making decisions aimed at improving the contribution of PMDs to ending the scourge of TB.

## Methods

### Study area

The study was conducted in Ebonyi State in the southeastern Nigeria. The state is divided into 3 geopolitical zones, with 13 local government areas (LGAs), 171 political wards, and 275 health wards and had a projected population of 3,132,8271 in 2019 [28]. It is mainly rural with about 75% of the population living in the rural areas. The main occupation of the people is farming but it has great potential for solid mineral exploitation and mineral-based industries.

There are 557 registered public and private health facilities in Ebonyi State and these include 2 tertiary health institutions, 13 general hospitals, 6 faith-based hospitals, 417 primary health care facilities and 119 private hospitals and clinics [29]. There are 148 DOTS facilities in the state together with other informal private health service providers such as PMDs, Traditional Birth Attendants and Traditional Bone Setters. There are 1843 PMDs registered with National Association of Patent and Proprietary Medicine Dealers (NAPPMED), the association of operators of patent medicine vendors in Ebonyi State.

### Study design and population

This was a cross-sectional qualitative study of PMDs and TB Control Programme Managers (TBCPMs).

### Inclusion criteria

All PMDs who were registered with Ebonyi State chapter of NAPPMED and all TBCPMs in the employ of Ebonyi State TB Control Programme were considered eligible. Those included have operated a patent medicine shop for at least 6 months for PMDs and served as TB programme manager for up to 6 months for TBCPMs.

### Sample size

Sample size was estimated based on the concept of ‘information power’ proposed by Malterud et al. [30] According to the model, the sample size in a qualitative study should be based on the sufficiency of the information derivable while considering the study aim, sample specificity, use of established theory, quality of dialogue, and analysis strategy. Sample sizes of 64 PMDs for eight Focus Group Discussions (FGDs) and 5 TBCPMs for five In-Depth Interviews (IDIs) were considered adequate.

### Sampling technique

Participants were selected through multi-stage sampling method. Firstly, Ebonyi North and Ebonyi Central geopolitical zones were selected by balloting. The LGAs in the selected zones were stratified into urban and rural areas and one LGA was selected in each stratum giving a total of four LGAs. In third stage, two NAPPMED branches were selected in each of the four LGAs. Finally, the chairperson of each selected NAPPMED branch was asked to purposively select eight PMDs who took part in FGDs. One most senior TBCPM serving at each of the selected LGAs and one serving at the state TB control office were selected to take part in IDIs. Participants were approached face-to-face, through telephone call and short message services (SMS).

### Study instruments

The study instruments included FGD and IDI guides developed by the research team. The FGD guide was designed to assess PMD’s knowledge and practices about TB and their involvement in TB control activities. The IDI guide was designed to evaluate TBCPMs’ perspectives on the involvement of PMDs in TB control, their perceived constraints to PMDs’ involvement and approaches for addressing those constraints so as to foster effective collaboration with them.

### Data collection

Eight FGD sessions comprising 8 PMDs each were held at secluded venues convenient to and close to the participants and lasted between 45 to 60 min each. Five IDI sessions were held with TBCPMs in their offices and lasted for 40–60 min each. All discussions and interviews were conducted in English Language, chosen at the discretion of the participants. Data collection was done using audio-recording device and backed up with note-taking. The FGDs and the IDIs were moderated by the researchers, assisted by four research assistants who acted as note-takers.

### Data analysis

Data were analyzed using thematic approach. The audiotapes were transcribed verbatim within 24-h and compared with the written notes. The transcription was checked against the audiotape for completeness and accuracy. Further, each script was checked against the audiotape by an independent reviewer. Coding of transcripts was done by two members of the research team based on predetermined and emergent themes. The data coders first familiarized themselves with the data through joint reviews and discussions. Thereafter, they assigned preliminary codes to the data and identified themes in the codes across the different group discussions and interviews. These themes were harmonized with the predetermined themes and new ones were named. Six themes of two predetermined and one new one from FGD and one predetermined and two new ones from IDI emerged from the study. The themes from the FGD included PMDs’ knowledge of TB, PMDs’ practices about TB and PMDs’ willingness to be involved in TB control while the themes from the IDI included perspectives of TBCPMs regarding involvement of PMDs in TB control, TBCPM’s perceived constraints to involvement of PMDs in TB control, and perceived approaches for addressing the constraints to involvement of PMDs in TB control.

## Results

### Participants’ profile

Eight FGD sessions were held with PMDs and 5 IDIs with TBCPMs. Each of the FGDs was made up of 8 PMDs giving a total of 64 participants with an age range of 23–50 years and median age of 32 years. In the urban area, there were 18 female and 14 male participants. In the rural area, there were 12 female and 20 male participants. Six of the participants comprising 4 males and 2 females were graduates including 2 who were also Junior Community Health Extension Workers; all the other participants completed secondary education. The duration of practice as PMD ranged from 2 to 14 years with a median of 7 years (Table 1).

**Table 1:** Sociodemographic characteristics of the participants

Variable	Frequency (%)	
	FGD^a^ participants n = 64	IDI^b^ participants n = 5
*Age range (years)*		
21–30	20 (31.3)	1 (20.0)
31–40	18 (28.1)	2 (40.0)
41–50	15 (23.4)	1 (20.0)
51–60	11 (17.2)	1 (20.0)
*Sex*		
Male	34 (53.1)	5 (100)
Female	30 (46.9)	0 (0)
*Education*		
Secondary	58 (90.6)	0 (0)
Post-secondary	6 (9.4)	5 (100)
*Location of work place*		
Rural	32 (50.0)	2 (40.0)
Urban	32 (50.0)	3 (60.0)
*Work experience (years)*		
0–5	26 (40.6))	3 (60.0)
6–10	20 (31.3)	0 (0)
11–15	18 (28.1)	1 (20.0)
> 15	0 (0)	1 (20.0)

^a^Focus group discussion

^b^In-depth interview

Five IDIs were held with the TBCPMs. Four of the discussants are Tuberculosis Supervisors at the LGA level while the fifth works as a TB Programme Manager at the state level. The age range of the discussants is 30–53 years with a median of 40 years. All the discussants are males and attained tertiary education. The work experience of the managers ranged from 3 to 27 years with a median of 4 years (Table 1).

### Patent medicine dealers’ knowledge of tuberculosis

Most of the participants were aware that TB is caused by a germ and could be transmitted from one person to another. A few of them were specific that bacterium is the cause of the disease and some of them even mentioned the name of the bacterium. The participants knew many of the signs and symptoms of TB. Most of them knew that chronic cough is an identifying criterion for TB with some of them even describing the disease as a chronic cough. These were captured thus:

“Tuberculosis is a bacterial infectious disease caused by Mycobacterial tuberculosis”. It can affect different organs of the body including lung, bones, and lymph nodes. It can also affect animals; the one that infects cow is called Mycobacterial bovis” (Group (G) 4, Number (N) 26, male PMD, urban area).

“I begin to suspect TB when my customer has cough that has lasted for about two weeks or more, night sweat, weight loss or coughing out of blood” (G7N53, female PMD, rural area).

“When someone affected with TB coughs, he introduces the germ into the air which can infect another person” (G8N64, male PMD, rural area).

However, few of the participants had poor knowledge of the disease, its symptoms and mode of transmission. A participant referred to TB as a venereal disease, explaining that it is transmitted through sexual intercourse and another participant mentioned that drinking water using same cup used by someone who has TB could make another contract the disease as captured in these quotes:

“Tuberculosis is a venereal disease. It is contracted through sexual intercourse between a man and a woman” (G5N38, male PMD, rural area).

“A person affected by TB should not drink water using the same cup as other members of his family to avoid infecting others with the disease” (G7N52, female PMD, rural area).

Two other participants who did not know the correct symptoms of TB were of the opinion that TB causes low temperature and one believed that TB affects only adults, explaining that children are rarely affected. These were presented as follows:

“The body of a person affected by TB swells up when the disease is advanced and the patient will have low temperature” (G6N45, female PMD, rural area).

“Tuberculosis causes low temperature and usually affects only adults; it is not common in children” (G4N28, female PMD, urban area).

### Patent medicine dealers’ practices about presumptive tuberculosis

Two discussants made it clear that they made referral of client with chronic cough a priority. One of them stated that he always refers his clients because he is duty-bound to do so and NAPPMED has penalty for any of her members who treats clients with serious condition beyond the limit permitted them by law. Another discussant said that she valued the lives of her clients more than the money she makes from her business and that patient referral is a noble thing to do. She believes that referring clients makes her gain the trust of the people because they will see her as someone who knows her limitations and does what is right. These were captured in the following quotes:

“Nothing will make me not to refer customers with chronic cough as it is binding on all our union (referring to NAPPMED) members to refer such customers. Our union has penalty for any member who attempts to treat what is beyond him or her to treat” (G7N55, male PMD, rural area).

“Life is more precious than money and referring customers with chronic cough will not decrease my income. It will even promote my business because the villagers will say that I am a good person who knows my limit and so bring more sick persons to me for treatment. It is noble to do so” (G1N6, female PMD, urban area).

However, the belief that they could treat any cough irrespective of the cause and duration of the symptom was evident in statements made by four other discussants, which was corroborated by few others. Their action is to first administer cough medicines to clients and observe them for a period ranging from two to seven days before considering to refer the clients. The participants based their decision regarding client referral on whether there is improvement symptomatically with the cough medicines or not; clients who did not improve with the medicines were referred while those who did were not. Two of the discussants had these to say:

“When I give patients cough medicine, I ask them to come back after two days. If the patients improve with the treatment, I will not refer them to DOTS facility because it shows that the medicine I have given them is good for the cough. If they come back and the sickness is still serious, I tell them to go to hospitals like Mile IV Hospital Abakaliki (a hospital popularly known for treatment of TB patients) or the Teaching Hospital in Abakaliki for treatment” (G6N50, female PMD, rural area).

“I give them medicines and ask them to take them for seven days after which I refer them to Mile IV Hospital Abakaliki if they do not improve with my treatment” (G3N18, male PMD, urban area).

Again, statements made by two participants which were corroborated by few others show that both the providers and the patients did not know that TB treatment is free of charge to patients. The providers did not educate the patients that treatment for TB is free of charge when patients complained of financial difficulty following attempts to refer them. These were captured thus:

“Occasionally, patients complain of financial constraints when I ask them to go to the hospital for treatment and they request that I give them cough medicines with the little money they have” (G2N10, male PMD, urban area).

“Sometimes, my cough clients are so poor that instead of asking them to go to hospital when I know they will not be able to afford the transportation and treatment and especially when they have requested that I treat them at my shop, I decide to treat them myself” (G8N57, male PMD, rural area).

Some of the participants alleged poor attitude of formal health facility staff and prolonged waiting time in hospitals, citing them as reasons why they do not refer their clients with chronic cough to health facilities. The discussants presented their reasons in a way that suggests that they were mere assumptions made based on feedbacks gotten from their clients. Two of the participants said:

“Occasionally, the health workers in hospitals feel less concerned when I refer patients to them; my clients usually complain of poor attention given to them and the preferential treatment given to some other persons. This poor attitude discourages me from referring clients sometimes” (G1N2, male PMD, urban area).

“Occasionally, patients’ complaints of prolonged waiting time and too many protocols involved in getting services in public health facilities makes them to refuse my referral and prefer to be treated in my facility” (G4N29, female PMD, urban area).

### Patent medicine dealers’ willingness to be involved in tuberculosis control

Most of the participants overtly expressed their willingness to get involved in TB case finding efforts, stating that they are ready to collaborate with the NTBLCP, at whatever level they are invited to be involved. One of the participants said:

“People living in this village know us (referring to PMDs) and they trust us. If government, individuals, Non-Governmental Organizations or other partners in TB programme want to look into this matter (referring to TB case finding), they should collaborate with us to pass down their message” (G3N20, male PMD, urban).

A participant made it known that PMDs could serve as linkage between clients with chronic cough and DOTS providers. Two other discussants went on to say that they can get involved in sputum sample collection from patients. Some of the participants had these to say:

“We can help in TB case finding effort by linking patients with chronic cough to the DOTS facility personnel” (G6N44, female PMD, rural area).

“When we see patients with chronic cough, we can collect information about them including their names, phone numbers and addresses and send to DOTS providers to follow them up” (G5N35, male PMD, rural area).

“We can help is in collecting sputum samples from patients whose cough has lasted long if we are given sputum cup to do so” (G8N59, male PMD, rural area).

Two participants suggested that there should be an official means of communication between PMDs and DOTS facility personnel to ensure there is effective communication between them. The participants said:

“We frequently encounter patients with long lasting cough. If there are means of communication between us and DOTS staff, it will allow for easy linkage of patients who present to us to DOTS centres” (G2N15, female PMD, urban area).

“Provide us with phone numbers for contact with DOTS providers; that way we will be able to work together by referring our customers to them” (G1N7, male PMD, urban area).

### Perspectives of TBCPMs regarding involvement of PMDs in tuberculosis control

All discussants attested to involvement of PMDs in the activities of TB control. A discussant cited TB Reach supported by German Leprosy and TB Relief Association (GLRA), as a programme in which PMDs were trained on TB case detection and encouraged to identify and refer PTBCs to DOTS facilities. The discussant had this to say:

“In the project TB Reach, GLRA trained about 10 PMDs in the state and requested them to be referring all PTBCs to the nearest DOTS facility. However, the project could not continue because there were no incentives for the PMDs” (N4 TBCPM, rural area).

The discussant further recalled how PMDs were invited to participate in workshops on TB and reiterated how their participation has served as a motivation to them because usually after the workshop and several years after, the co-operation of the PMDs with the TB control programme was remarkable; this was evidenced by the increase in number of referrals of PTBCs by PMDs. He said:

“Previously, PMDs used to be invited to workshops on TB by the NTBLCP after which they were given some rewards. This motivated them and till today, some of the PMDs who participated in those workshops still call me each time they have PTBCs” (N4 TBCPM, rural area).

A second discussant narrated how the NTBLCP, through the TBCPMs, has been engaging PMDs in the form of TB awareness creation and sensitization during PMDs’ monthly meetings, encouraging them to screen their clients who present with cough for TB, by asking them for the duration of the symptom and other symptoms, and referring those eligible to DOTS centres for diagnosis and treatment. Another discussant corroborated the second but alleged poor cooperation of the PMDs with NTBLCP, stating that PMDs have not been referring clients with much interest. The narration and allegation were captured thus:

“We (referring to TBCPM) usually organize visits to create an awareness during the monthly PMDs’ meetings. We engage them by taking few minutes to create awareness about TB and its signs and symptoms and educate them on the procedures of referring PTBCs to DOTS centres for diagnosis” (N2 TBCPM, urban area).

“When we attend their meetings, we educate them on signs and symptoms of TB. Afterwards we encourage them to screen clients and send those eligible to DOTS facilities closest to them by issuing them with sputum request forms with which they could refer PTBCs. However, they have not been doing that with much interest” (N3 TBCPM, rural area).

A discussant alleged that there is on-going malpractice in management of TB in patent medicine shops, stressing that engaging PMDs more formerly in TB control could help to reduce the malpractice level. He gave the following narrative as a proof of what happens:

“I usually stand by the side of a drug vendor in the evening to take a look at the population of clients that go to patent medicine shops. Occasionally, I pretend I am buying medicine when in actual sense I am there to observe how the PMDs do their clerking and how they treat the patients. Usually, I feel disappointed where very severely ill persons will come to PMDs who are usually semi-illiterate and after listing his complaints, the PMDs will claim to have diagnosed the problem and start mixing medicines for the patients as if they are professors of medicine. So, it is important to formally involve PMDs in TBLCP to avoid this kind of problem by sensitizing them, orientating them and possibly giving them enlightenment because if you leave them, they will do worse things” (N1 TBCPM, urban area).

Two other discussants also alleged mismanagement of TB patients by PMDs, stating that PMDs give antibiotics for treatment of cough without knowing the implications. The discussants condemned the use of antibiotics by the PMDs in the treatment of cough because there is a specified national guideline for treatment of TB which the PMDs are not observing and treatment of TB with wrong antibiotics or with anti-tuberculosis in inadequate dose predisposes to development of resistant organisms. One of the discussants expressed his views this way:

“Patent Medicine Dealers have patients that receive antibiotics for more than a month without knowing the implication in the emergence of drug resistant TB. The practice is bad because some of the antibiotics sold by PMDs are also used to treat TB and using it for a shorter duration than is prescribed in the national treatment guideline can lead to development of antibiotic resistance” (N5 TBCPM, rural area).

### Tuberculosis control programme managers’ perceived constraints to involvement of PMDs in tuberculosis control

Most of the discussants complained of poor co-operation of the PMDs with the TB control programme in terms of referral of PTBCs. The discussants identified constant demand for incentives for the work of referring PTBCs by the PMDs as the major constraint to involvement of PMDs in the control programme. Two of the discussants noted:

“The constraint to involvement of PMDs is the constant expectation for incentives either financial or in the form of entertainment when we engage them in their meetings, without which they may not give us audience or full attention to the sensitization and may not do what we ask of them” (N5 TBCPM, rural area).

“The PMDs are very reluctant in carrying out PTBC referral activity because there is no money involved in doing so. They want something that is symbiotic and can’t operate without give and take policy” (N1 TBCPM, rural area).

Another discussant narrated his experience with the PMDs which show that they do not co-operate with TB control programme when no incentives are provided to them. He said:

“Each time I attend PMDs’ meeting, they will be paying attention to me based on what they can get from me thereafter and if at the end of the day I did not present anything that they will benefit instantly from, they will just do away with whatever I would have taught them except very few of them” (N2 TBCPM, rural area).

Another constraint to involvement of the PMDs perceived by a discussant is the ineffective payment system for personnel involved in sputum sample collection and transportation to laboratory. The discussant explained that inadequacy of fund for transportation of sputum sample from collection site to laboratory for diagnosis and difficulty in accessing the fund, when it is available, is a problem not only for the personnel working with NTBLCP but also in involving PMDs. Such difficulty will delay sputum sample collection and transportation to laboratory. Narrating his experience as a manager in the NTBLCP, the discussant said:

“A barrier to effective transportation of sputum samples is the under-payment of personnel involved. This happens because personnel involved in sputum transport usually makes several visits, say five or six, to DOTS facility for sputum sample collection, submission and collection of result but usually gets paid for only two or three of such visits especially when the result is negative. Some of our health workers and DOTS focal persons who are supposed to be doing this work more than the PMDs are tired because the system of payment is very difficult to cope with. This challenge is not what any of us at the grass root, including the State TB Control Officer, can easily overcome but the funders of the programme” (N4 TBCPM, rural area).

However, a discussant made it known that the NTBLCP has earmarked money for movement of sputum specimen and stated that for every case that is referred which is presumptive, the programme pays the PMD involved. The participant had this to say:

“A fund has been earmarked for movement of sputum specimen from the facility to Gene-Xpert sites. For intra-city referral, the budget is that if a PMD moves patient’s sputum specimen within an LGA, he will be entitled to #1500.00 ($3.14) and if it is between two LGAs, he will be entitled to #3000.00 (#6.28) and PMDs will be permitted to move samples twice a week” (N1 TBCPM, urban area).

### Perceived approaches for addressing the constraints to involvement of PMDs in tuberculosis control

The opinion of most of the TBCPMs is that PMDs should be more deeply engaged in NTBLCP by first imparting more knowledge of the disease to them through orientations, trainings, and continuing education. To achieve this purpose, two of the discussants suggested that the already existing TB awareness creation and sensitization of PMDs during their regular monthly meetings should be strengthened. The participants made their opinions known with these comments:

“The first thing in addressing constraints to the involvement of PMDs is to start imparting knowledge of TB on them by organizing trainings so they will have the knowledge because TB is not like other diseases that can be treated with usual prescriptions. The PMDs will be made to know the risks to themselves and to the communities of mismanaging TB cases and the likely outcomes of good management of cases. After imparting the knowledge on them, we have to work with them; some of the PMDs can be used as DOTS personnel under strict monitoring of LGA programme managers” (N2 TBCPM, rural area).

“If PMDs are sensitized well, they will be able to screen, identify and refer cases to DOTS centres. They may also be given the opportunity to collect samples and send to the laboratory and can also be involved in treatment of cases by serving as treatment supporters. For those who will be able to do these, the programme will provide them with recording and reporting materials” (N5 TBCPM, rural area).

Another discussant suggested that any measures to improve the cooperation of the NTBLCP with the PMDs should take into account the business interest of the drug vendors. He put it this way:

“The best thing to do is to engage PMDs and give them an idea of how they can technically refer chronic cough patients without losing their prestige, because they are highly regarded by members of the communities where they operate. That is a project that will take into cognizance their own business interest and also public health interest” (N1 TBCPM, urban area).

A discussant recommended that the funders of NTBLCP should accommodate a budget line to support communication between PMDs and DOTS service providers on one hand and the PMDs and PTBCs on the other hand in order to facilitate the referral and linkage of clients with DOTS service. The recommendation was captured thus:

“Effective communication between PMDs and PTBCs on one hand and between PMDs and DOTS personnel on the other hand is necessary. The PMDs should collect the phone numbers of every PTBC seen and communicate with TBCPM and DOTS staff to ensure that such patient can easily be tracked. The funders of TB control programme should accommodate a budget line to support the PMDs in the communication process, providing them with airtime to facilitate the linkage of patients to DOTS centres and vice versa” (N3 TBCPM, rural area).

Another discussant made it known that there is hope for funding of involvement of PMDs in TB control in the new model for utilization of Global Fund, stating that there is an Independent Principal Recipient of the Fund for private sector on TB control. Funding of PMDs’ involvement in TB control could partly be taken care of with the fund for private sector on TB. The participant had this to say:

“There is an independent principal recipient for the Global Fund purely for private sector. The principal recipient will focus purely on private sector on TB control while the National TB Control Programme will focus on the public sector. Involvement of PMDs in TB control could be partly funded with the fund for private sector on TB” (N1 TBCPM, urban area).

A discussant, who alleged that there is unhealthy relationship between the PMDs and the pharmacists in Nigeria due to what he referred to as ‘the policing aspect of controlling the PMDs by the inspectorate division of pharmacy, the Pharmacist Council of Nigeria (PCN)’, advised that the PCN should be supportive in its supervision of the PMDs. This is because hostile regulatory approach causes a lot of harm to TB control and the health system at large. The discussant further sued for the incorporation of PMDs into the formal health system to take advantage of their wide reach-out in the communities. These are captured in the following quote:

“The way forward is to get the PMDs close, sit down and dialogue with them so that they will know their limit in treatment of patients. When they reach their limit, they should refer the patients to the next level of health care delivery. The health sector will be deceiving herself if it does not incorporate PMDs into the formal health system because any village you go to and you do not find PMDs, there is really no human beings there. They are the most established healthcare provider in terms of reach out and this is because their way of operation suits the poverty level of the people. Let the pharmacy department of the Ministry of Health be friendly with PMDs. Let there be no policing aspect of controlling the PMDs by the PCN because such regulatory approach is destroying things in the health sector” (N1 TBCPM, urban area).

## Discussion

This study investigated the constraints to involvement of PMDs in TB control and the perceived approaches to address such constraints. We found knowledge gaps about TB and its control among PMDs, amidst constant demand for incentives by the PMDs and inability of NTBLCP to keep up with such demands as major constraints to effective involvement of PMDs. A more robust engagement of PMDs by NTBLCP through improved TB care knowledge and motivation are perceived solutions.

Despite that most of the PMDs demonstrated good knowledge of TB, the lack of knowledge of signs, symptoms and mode of transmission of TB suggests that they may not be able to identify PTBCs to make necessary referrals to DOTS facility. Of particular concern is the lack of knowledge of free treatment policy for TB among some of the participants. This means that those PMDs may not be able to educate their clients properly and this may negatively affect clients’ search for TB care. Again, despite the claim by few participants that they treated patients with chronic cough because of patients’ complaints of financial difficulty not being confirmed since the participants were speaking for the patients, ignorance of free treatment policy for TB in the communities could negatively affect peoples’ seeking behaviour for TB care. This underscores the need for strengthened public sensitization about free treatment for TB to increase their health-seeking behaviour. Lack of affordability due to financial barrier is a recognized factor that negatively influences health-seeking behaviour [31, 32].

Although the reported claims that clients’ financial difficulty, poor attitude of staff and prolonged waiting time in public health facilities are the reasons for non-referral of clients with chronic cough by the PMDs were also not confirmed, they indicate that the participants have a poor understanding of the modus operandi of the NTBLCP in Nigeria. Under the NTBLCP, care for TB is given by designated trained staffs whose responsibilities are to attend to patients promptly and at all times at no financial costs to the patients. The claims by our participants may be true in situations where the search for health service is for other disease conditions; for instance, in a previous study on malaria, participants mentioned difficulty in accessing treatment at public health facilities, ability to purchase affordable drugs and short waiting time before treatment as the main reasons for using PMDs for their malaria treatment needs [33]. Client and providers’ lack of knowledge of TB control programme has previously been reported as a cause of delay in seeking care for symptoms suggestive of TB [34]. Our finding calls for a need to fill up the existing TB control knowledge gap through public education and sensitization in order to properly orientate the people.

Most of the participants treated chronic cough patients for too long and considered referral when treatment they had given the patients failed to improve their conditions. This approach was also reported among PMDs in a previous study [35]. This type of treatment is empirical since there is no laboratory confirmation of diagnosis. The time interval between first contact of a patient with chronic cough and diagnosis and commencement of appropriate medicines is critical to successful treatment of TB. This interval should be limited as much as possible to prevent the advancement of the disease and development of complications. The penchant to treat cough patients for too long before considering referral is a dangerous trend that should be discouraged at all levels of health service delivery. Even with improvement with medicines given, chronic cough clients should be referred to DOTS facilities thereafter and without delay, for screening, diagnosis and appropriate treatment. Empirical treatment, which is employed by trained health care professionals using clinical knowledge of signs and symptoms of diseases and of drugs, is beyond the knowledge and skills of PMDs. The drug vendors have been reported to have poor knowledge of health care and drugs [10], often dispense drugs improperly [10, 16, 36], and provide services beyond their legal scope of practice [10].

Empirical treatment of chronic cough, if improperly employed, may result in the risk of misdiagnosis of severe conditions, delay in diagnosis, increased cost of care and where antibiotics with anti-TB activity are inappropriately used for the treatment of PTBCs, which has previously been reported among PMDs [6, 26, 27, 36, 37], it could promote emergence of antibiotic resistant micro-organisms [38]. Empirical treatment of patients with chronic cough should be used with caution, and serious conditions including TB, bronchial malignant tumors and other pulmonary diseases should be ruled out [39].

The overt expression of willingness to collaborate with NTBLCP by most of the PMDs is welcome. A previous study in Nigeria also reported that most of the PMDs were positively disposed to playing roles in TB control to increase case-detection and indicated interest to cooperate with the NTBLCP if they were trained [8]. The disposition of the PMDs lends credence to the pressing need for training of the PMDs on TB care. Patent Medicine Dealers are plentiful in many parts of Nigeria where their shops had been described as being more than public and private health-care facilities [40], and may be more accessible [16]. This show of willingness for collaboration with NTBLCP is an opportunity that should not be neglected. Involving PMDs in TB control will help create a sense of belonging among them, nurture mutual understanding and healthy relationship and foster the much-needed collaboration of PMDs with NTBLCP to increase TB case finding.

Among the constraints perceived by the TBCPMs, the need for sustained incentives for the PMDs was prominent. This was not unexpected since effective client referral and contact tracing demands making some time and financial commitments especially in the areas of communication and transportation. Being profit-oriented private business men and women who rely on the proceeds from their business for their daily needs and those of their dependents, PMDs may not have the sustained commitment needed for rendering services that speak to the goal of ending scourge of TB and other public health activities if they are not motivated. The need for motivation has been demonstrated by Uguge et al. who reported TB case yield of 9% from PTBCs identified by PMDs out of a total yield of 11% from all presumptive clients screened in an intervention carried out in Nasarawa State, north central Nigeria [41].

The reported level of involvement of PMDs by the TBCPMs was neither encompassing nor regular. Rather than give them only PTBC referral forms, the PMDs can be trained on the process of sputum sample collection and empowered to collect samples with sputum cups deployed to them and arrangement made for the DOTS personnel to pick up the samples. Interestingly, some of the PMDs made it known that they are willing to get involved in sputum sample collection and requested that they should be provided with sputum cups to aid in sputum sample collection. If implemented, this will reduce missed opportunities with PTBCs and facilitate diagnosis and treatment. This was the case in Bangladesh with drug vendors and the output was great [42]. This was also the case in India where a three-year intervention project that engaged unqualified rural informal health care providers, akin to PMDs in Nigeria, in TB control led to remarkable output with referral of 1578 PTBCs and detection of 194 TB cases [43]. This huge contribution was largely facilitated by use of mobile Health (mHealth) technology through which 67% of the PTBCs referrals and 65% of the TB case detection were made. Patent Medicine Dealers’ engagement and with mHealth strategy is a good option for Nigeria with widespread and increasing use of internet services through mobile phones and social media [44]. The mHealth strategy will hopefully address some of the constraints identified in this study by improving PMDs’ TB knowledge, PTBC referral and communication with DOTS facility personnel on one hand and PTBCs on the other.

Patent Medicine Dealers may also serve as treatment supporters to patients on DOTS therapy and with their spread in the community, they may be useful in tracing contacts of TB patients. Their active engagement as treatment supporters could increase adherence, treatment completion and cure rate.

The alleged poor relationship existing between the PMDs and the pharmacy division of the formal health sector calls for concerted effort at improvement especially the apparent policing aspect of the Pharmacy Council of Nigeria (PCN) over the activities of the PMDs. Similar to this finding, poor collaboration with the pharmacists was a common opinion expressed by PMDs in a previous study conducted in Kwara State, north central Nigeria [45]. It was reported that all the PMDs who had shops were registered members of NAPPMED but none was licensed by the PCN due to rivalry and lack of collaboration between them. In another study, it was reported that 81.4% of drug vendors in 16 states of Nigeria were registered with the NAPPMED whereas only 13.4% were registered with the PCN [40], a body that is supposed to register and regulate the activities of the drug vendors. These findings suggest that the PCN may not be adequately regulating the activities of the PMDs and this is not good for the country’s health system. Rather than promote a good working relationship with these critical players in the informal sector of the country’s health system, a hostile regulatory approach or partial registration and regulation of the PMDs has the potential for causing harm to the Nigerian public health system especially in the area of TB control.

Provision of health care in Nigeria is largely private sector driven [15], and every approach used for enforcement of regulatory standards in the sector must not only be legal but should be fair to the actors in the field through application of supportive supervisory approach. Co-operation between the NAPPMED and the PCN is required to develop mechanisms that both raise drug vendors’ standards and facilitate regulatory compliance through increase in drug vendors’ registration with the PCN [40]. Continuing education of PMDs through trainings, workshops and seminars, rather than make them overstep the limit permitted them by law, will help to fill their knowledge gap and make them to realize the type of services they are permitted to provide and when it becomes necessary to refer patients to higher level of care. The fact that NAPPMED penalizes her members that treat clients with serious conditions beyond the limit permitted them by law is a desired practice that indicates the association’s resolve to comply with regulatory standards. This good practice should not only be promoted by NAPPMED but also by PCN and other health regulatory authorities at various levels of care to discourage management of patients with chronic cough at patent medicine shops. Looking beyond rendering TB care services, PMDs are indispensable to the delivery of other health services needed for achieving Universal Health Coverage (UHC) in Nigeria and other low- and middle-income countries with shortage of human resources for health. This is due to their wide spread in the communities, their acceptability and huge patronage by people especially in hard-to-reach rural areas where health service coverage by the formal sector is inadequate. Their potential in giving a boost to health service delivery underscores the pressing need for the government and health regulatory authorities to build and maintain a healthy relationship with the PMDs.

### Limitation of the study

The researchers had no control over the selection of FGD participants. The study findings may not be generalized to the entire PMDs in Ebonyi State because of non-random selection of participants by NAPPMED chairpersons who may have been biased in their selection process. However, selection bias was reduced by use of multi-stage sampling method in selection of geopolitical zones, LGAs and NAPPMED branches and application of set criteria including being an executive member and length of membership of the association.

## Conclusions

This study found some knowledge gaps about TB signs, symptoms, transmission pattern and its control including free-treatment policy for TB patients among PMDs. Both PMDs and TBCPMs are willing to collaborate in TB control efforts but inability of the NTBLCP to keep up with constant demands for incentives by the PMDs for referral of PTBCs and inadequate communication between the two parties constitute constraints. We recommend continuing TB education, awareness creation and sensitization for the PMDs and support for formal communication between the PMDs and the DOTS service providers on one hand and PMDs and the PTBCs on the other as a means of facilitating linkage of the PTBCs to DOTS services to increase TB case detection. The NTBLCP should establish a channel of communication with the PMDs through a toll-free line for linkage of PTBCs to DOTS programme personnel and to facilitate patient tracking and contact tracing. This will encourage PMDs’ participation in TB control, reduce delay in TB diagnosis and treatment and increase survival of TB patients.

## Data Availability

All relevant data collected during this study are presented in this paper. Additional data could be available upon request to the corresponding author.

## Abbreviations

DOTS: Directly observed treatment short-course
FGD: Focus group discussion
GLRA: German Leprosy and TB Relief Association
IDI: In-depth interview
LGA: Local government area
mHealth: Mobile health
NAPPMED: National Association of Patent and Proprietary Medicine Dealers
NTBLCP: National Tuberculosis and Leprosy Control Programme
PCN: Pharmacy Council of Nigeria
PMD: Patent medicine dealer
PTBC: Presumptive tuberculosis client
TB: Tuberculosis
TBCPM: Tuberculosis control programme manager
TBLS: Tuberculosis and leprosy supervisor
UHC: Universal health coverage
WHO: World Health Organization
